# Evaluación comparativa de la vigilancia en salud pública de COVID-19 en Colombia: primer semestre

**DOI:** 10.7705/biomedica.5812

**Published:** 2020-11-15

**Authors:** Alexandra Hurtado-Ortiz, José Moreno-Montoya, Franklyn E. Prieto-Alvarado, Álvaro J. Idrovo

**Affiliations:** 1 Departamento de Salud Pública, Escuela de Medicina, Universidad Industrial de Santander, Bucaramanga, Colombia Universidad Industrial de Santander Departamento de Salud Pública Universidad Industrial de Santander Bucaramanga Colombia; 2 Subdirección de Estudios Clínicos, Fundación Santa Fe de Bogotá, Bogotá D.C., Colombia Universidad Nacional de Colombia Fundación Santa Fe de Bogotá BogotáD.C Colombia; 3 Dirección de Vigilancia y Análisis del Riesgo en Salud Pública, Instituto Nacional de Salud, Bogotá D.C., Colombia Instituto Nacional de Salud BogotáD.C Colombia

**Keywords:** infecciones por coronavirus, epidemias, notificación de enfermedades, monitoreo epidemiológico, distribuciones estadísticas, Colombia, Coronavirus infections, epidemics, disease notification, epidemiological monitoring, statistical distributions, Colombia

## Abstract

**Introducción.:**

La vigilancia en salud pública y las decisiones sanitarias recomendadas son fundamentales para el manejo adecuado de la pandemia de SARS-CoV-2.

**Objetivo.:**

Hacer una evaluación comparativa del desempeño de los departamentos colombianos de este atributo del sistema de vigilancia con base en la calidad de los datos y construir la clasificación nacional según el desempeño.

**Materiales y métodos.:**

Se analizaron los casos acumulados publicados por el Instituto Nacional de Salud entre el 6 de marzo y el 1° de septiembre de 2020. Para la comparación, los análisis consideraron el día en que se diagnosticó el primer caso como la primera fecha de análisis de cada departamento. El cumplimiento de la ley de Benford se evaluó con los valores de p en las pruebas de razón del logaritmo de la verosimilitud o ji al cuadrado. Se completó el análisis del atributo de calidad del dato con la letalidad observada en cada departamento, y se estableció la clasificación según el desempeño.

**Resultados.:**

La ciudad de Bogotá y el departamento del Valle del Cauca tuvieron un desempeño óptimo en la vigilancia en salud pública durante todo el periodo observado. Los datos sugieren que los departamentos de Antioquia, Nariño y Tolima tuvieron una buena contención y una adecuada vigilancia en salud pública después de la apertura económica iniciada el 1° de junio de 2020.

**Conclusión.:**

Se obtuvo una clasificación de los departamentos y de Bogotá según la calidad de los datos de vigilancia en salud pública. Los mejores cinco entes territoriales pueden ser casos de estudio para determinar los elementos asociados con el buen desempeño.

## Abstract (en)

**Introduction:**

Public health surveillance together with good sanitary decisions is essential for the proper management of the SARS-CoV-2 pandemic.

**Objective:**

To compare the performance of Colombian departments based on the quality of the data and to build the national ranking.

**Materials and methods:**

We analyzed the accumulated cases published between March 6 and September 1, 2020, by the *Instituto Nacional de Salud.* To achieve comparability, the analyses considered the day the first case was diagnosed as the first analysis date for each department. The fulfillment of Benford's law was assessed with p-values in the log-likelihood ratio or chi-square tests. The analysis was completed with the lethality observed in each department and then the performance ranking was established.

**Results:**

Bogotá and Valle del Cauca had optimal public health surveillance performance all along. The data suggest that Antioquia, Nariño, and Tolima had good containment and adequate public health surveillance after the economic opening beginning on June 1, 2020.

**Conclusion:**

We obtained the ranking of the departments regarding the quality of public health surveillance data. The best five departments can be case studies to identify the elements associated with good performance.

La vigilancia en salud pública, además de las decisiones sanitarias nacionales, departamentales y municipales, son los elementos fundamentales de la respuesta a la pandemia de SARS-CoV-2. En la ciudad de Bogotá, la principal decisión adoptada fue el confinamiento generalizado en los hogares a partir del 20 de marzo de 2020 como un "simulacro obligatorio" decisión que fue implementada por el Gobierno Nacional del país desde el 24 de marzo de 2020 ^1^. Si bien en el lenguaje cotidiano se usó más la palabra *cuarentena,* es más adecuado usar la palabra *confinamiento,* dado que se encerraron personas sanas (cuarentena) y personas diagnosticadas con SARS-CoV-2 (aislamiento) ^2^. El confinamiento sirve para evitar la transmisión de una enfermedad infecciosa y permite ganar tiempo para mejorar la respuesta ante una epidemia; esto último implica mejoras en la vigilancia en salud pública, en la capacidad de hacer pruebas y en el número de camas hospitalarias y unidades de cuidados intensivos para el caso de la pandemia de COVID-19.

En Colombia, el primer caso de SARS-CoV-2 se notificaron en Bogotá el 6 de marzo de 2020 ^3^ y a partir de ahí se reportaron casos en diversos lugares del país, configurándose, así, una pandemia con manifestaciones heterogéneas en las regiones. En unos lugares, después del diagnóstico del primer caso se presentó rápidamente la transmisión comunitaria, y en otros, brotes dispersos, lo que implicaba momentos diferentes de inicio del incremento de casos que fueron modificándose según las actividades propias de cada lugar y su relación con otras regiones. En general, se puede decir que hay dos hitos fundamentales para entender la pandemia en cada departamento: por un lado, está el día del primer caso diagnosticado, que es variable, y, en segundo lugar, el día de levantamiento del confinamiento nacional, es decir, el 1° de junio de 2020. Por ello, después de seis meses de pandemia hay lugares donde ya pasó el primer "pico", otros que están en ese "pico" y otros que van subiendo la pendiente hacia el "pico" ^4^^-^^6^. Las directrices nacionales han seguido más estrechamente el comportamiento de las regiones donde más población afectada y que corresponden a la situación general del país.

En un estudio previo se presentaron los resultados de la evaluación del desempeño del sistema de vigilancia en salud pública nacional durante los primeros 50 días de pandemia, pues en ese momento los datos no eran suficientes para hacer análisis regionales. El análisis se basó en el cumplimiento de la ley de Benford, que ha demostrado ser útil para este tipo de evaluaciones durante las epidemias ^7^^-^^10^ (Idrovo AJ, Manrique-Hernández EF, Fernández-Niño JA. COVID-19 data quality in Brazil: Between applied science and politics. En prensa). Se observó que en la mayoría de los días la calidad de los datos fue buena ^11^. La acumulación de datos que ha tenido lugar en el proceso de vigilancia en salud pública durante el avance de la pandemia ha permitido la desagregación de los análisis a nivel regional.

En este estudio se presentan los resultados de una evaluación comparativa del desempeño de los departamentos y la ciudad de Bogotá, con el fin de determinar los casos más exitosos, que pueden servir de modelo a los demás.

## Materiales y métodos

Se hizo una evaluación comparativa (conocida como *benchmarking)*^12^ del desempeño de los 32 departamentos colombianos y del distrito de Bogotá, D.C. Los datos analizados corresponden a los que el sistema oficial de vigilancia en salud pública de Colombia genera como datos abiertos, y que se encuentran disponibles en https://www.ins.gov.co/Noticias/Paginas/Coronavirus.aspx. Si bien la información pública tiene varias variables, las usadas en este análisis incluyeron los datos de los casos acumulados entre el 6 de marzo y el 1° de septiembre de 2020 (180 días) sometidos a un proceso de evaluación basado en la ley de Benford, y los datos de letalidad. La lógica subyacente a la evaluación planteada es que para tener datos de calidad se deben hacer bien todos los procesos involucrados en las labores de notificación y de búsqueda activa por los equipos de respuesta inmediata, así como el procesamiento de muestras en el laboratorio y la generación de los reportes situacionales que se divulgan a nivel nacional.

**Figura 1:**
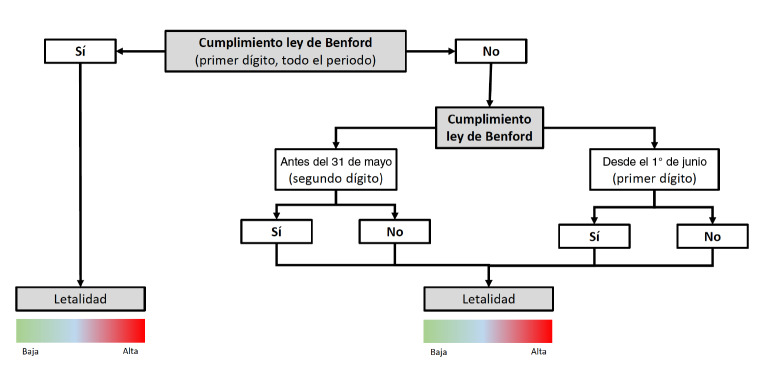
Algoritmo propuesto para evaluar el desempeño de la vigilancia en salud pública de los territorios colombianos durante los primeros seis meses de la pandemia

En la figura 1 se resume el algoritmo usado para la evaluación. El primer paso consistió en evaluar si se cumplía la ley de Benford para los primeros dígitos de los datos acumulados para todo el periodo analizado. Si esta se cumple, se puede inferir que se trata de entidades territoriales con un buen desempeño de la calidad del dato de la vigilancia en salud pública. En este grupo se estableció luego un orden según la letalidad (razón de fallecidos por casos diagnosticados como positivos), clasificando como mejor la entidad con menor fatalidad.

Cuando no se cumple la ley de Benford para el primer dígito durante todo el periodo, es posible que la contención de la epidemia se haya logrado, por lo que los casos nuevos podrían incrementarse a un ritmo mucho menor. Por ello se decidió explorar el cumplimiento de la distribución del segundo dígito de los casos acumulados hasta el 31 de mayo, último día antes de la primera apertura económica o levantamiento del confinamiento; además, se exploró el cumplimiento de la ley de Benford del primer dígito entre el 1 ° de junio y el 1 ° de septiembre.

De esta manera, si se cumple la distribución en ambos casos, es posible concluir que ha habido un buen desempeño de la calidad del dato de la vigilancia en salud pública y también es posible definir un posicionamiento basado en la fatalidad.

### La ley de Benford

Esta ley matemática, también conocida como "ley de Newcomb-Benford" o "ley de los números anómalos" ^13^, plantea que, en un conjunto de números, los que ocupan las primeras posiciones siguen una distribución conocida. La distribución esperada, según la ley de Benford, arroja un 30,103 % para el uno, 17,609 % para el dos, 12,494 % para el tres, 9,691 % para el cuatro, 7,918 % para el cinco, 6,695 % para el seis, 5,799 % para el siete, 5,115 % para el ocho y 4,576 % para el nueve. Los datos empíricos han demostrado que la frecuencia en que ocurren muchas situaciones de la naturaleza es una función inversa de su tamaño, por lo que los objetos más pequeños ocurren mucho más frecuentemente que los grandes ^14^^,^^15^.

Algo similar se ha observado para los demás dígitos, siendo más útil para fines prácticos el segundo, que permite una mayor variabilidad de los datos ^16^. La distribución esperada en este caso indica un 11,968 % para el cero, seguido de 11,389 %, 10,882 %, 10,433 %, 10,031 %, 9,668 %, 9,337 %, 9,035 % y 8,757 %, para los dígitos de uno a nueve sucesivamente. En estudios previos, el cumplimiento de la ley de Benford para el segundo dígito ha servido como indicador de la calidad de los datos en las endemias ^8^, lo cual podría extrapolarse a las condiciones de una epidemia en condiciones de contención.

### Métodos estadísticos

Para evaluar el nivel de cumplimiento de la distribución descrita por la ley de Benford en los datos observados (casos diarios acumulados), se realizaron, en orden, los siguientes análisis: primeros dígitos de casos acumulados en todo el periodo de observación mediante la prueba de razón del logaritmo de la verosimilitud ^17^. Dado que la contención real puede generar un estancamiento o un aumento muy bajo en la aparición de casos, se hizo un análisis con datos hasta el 31 de mayo usando la prueba de razón del logaritmo de la verosimilitud para los segundos dígitos. Por último, es posible que a partir de la apertura económica se iniciara un crecimiento acorde con la ley de Benford, lo que se evaluó con la prueba de ji al cuadrado ^18^ para analizar cada uno de los primeros dígitos de los datos posteriores al 1° de junio de manera independiente. Para ello se utilizó la macro *digdis* desarrollada por Ben Jann (ETH, Zurich) en el programa estadístico Stata 14™ (Stata Corporation, USA).

## Resultados

El desempeño nacional de la calidad del dato de la vigilancia, incluidos todos los casos acumulados hasta el día 180 de la pandemia, fue adecuado (p=0,44, prueba de razón del logaritmo de la verosimilitud) y siguió la tendencia que ya se había observado ^11^. La heterogeneidad de los entes territoriales se puede apreciar en el cuadro 1, en el cual se resume el cumplimiento de la ley de Benford enfocada en el primer dígito. Para hacer comparables los entes territoriales, se comenzaron a contar los días para el análisis de todos ellos a partir de aquel en que se diagnosticó el primer caso en cada territorio. Allí se puede apreciar que los entes territoriales han enfrentado la pandemia en diferentes periodos: algunos desde marzo y otros un par de meses después. Dieciséis de los entes territoriales cumplieron con la ley de Benford al inicio, lo cual sugiere un desempeño inicial adecuado en la calidad del dato, y Bogotá y el Valle del Cauca han mantenido un adecuado desempeño durante todo el periodo analizado.

**Cuadro 1:** Cumplimiento de la ley de Benford (primer dígito)* de los reportes de casos de COVID-19 en Colombia (casos acumulados) en diferentes momentos de la pandemia en cada territorio

Entidad territorial	Días desde el primer caso reportado																	
	10	20	30	40	50	60	70	80	90	100	110	120	130	140	150	160	170	180
Amazonas	0,05	0,13	0,22	0,02	<0,01	<0,01	<0,01	<0,01	<0,01	<0,01	<0,01	<0,01	<0,01					
Antioquia	<0,01	0,03	0,08	0,41	0,29	<0,01	<0,01	<0,01	<0,01	<0,01	<0,01	<0,01	<0,01	<0,01	<0,01	<0,01	<0,01	
Arauca	<0,01	0,03	0,08	0,41	0,29	<0,01	<0,01	<0,01	<0,01	<0,01	<0,01							
Atlántico	0,08	0,25	0,07	0,33	0,77	0,40	0,44	0,87	0,39	0,52	0,53	0,40	0,54	0,45	0,02	0,01		
Bogotá	0,07	0,14	0,61	0,75	0,17	0,43	0,57	0,84	0,99	0,78	0,49	0,74	0,82	0,91	0,99	0,81	0,30	0,31
Bolívar	0,17	0,50	0,65	0,43	0,23	0,30	0,60	0,22	0,15	0,06	0,67	0,85	0,78	0,39	0,07	0,13	0,04	
Boyacá	0,06	0,07	<0,01	<0,01	<0,01	<0,01	<0,01	<0,01	<0,01	<0,01	<0,01	<0,01	<0,01	<0,01	<0,01	<0,01		
Caldas	0,67	0,02	<0,01	0,14	0,09	0,05	<0,01	<0,01	<0,01	<0,01	<0,01	<0,01	<0,01	<0,01	<0,01	<0,01	<0,01	
Caquetá	<0,01	<0,01	<0,01	<0,01	<0,01	<0,01	<0,01	<0,01	<0,01	<0,01	<0,01	<0,01	<0,01					
Casanare	<0,01	0,01	<0,01	<0,01	<0,01	<0,01	<0,01	<0,01	<0,01	<0,01	<0,01	<0,01	<0,01	<0,01	<0,01	<0,01		
Cauca	<0,01	<0,01	<0,01	<0,01	<0,01	<0,01	0,09	0,07	0,08	0,02	0,11	0,27	0,39	0,24	0,09	0,12		
Cesar	0,34	0,02	<0,01	<0,01	<0,01	<0,01	<0,01	<0,01	<0,01	<0,01	<0,01	<0,01	<0,01	<0,01	<0,01	<0,01		
Chocó	0,01	<0,01	<0,01	0,02	<0,01	<0,01	0,08	0,02	<0,01	<0,01	<0,01	<0,01	<0,01	<0,01				
Córdoba	0,07	0,01	<0,01	<0,01	<0,01	<0,01	<0,01	<0,01	<0,01	0,01	0,02	<0,01	0,06	0,08	0,11			
Cundinamarca	0,50	0,09	0,52	0,32	0,05	<0,01	0,01	0,29	0,41	0,16	0,01	0,01	0,01	0,29	0,52	0,32	0,10	
Guainía	<0,01	<0,01	<0,01	<0,01	<0,01	<0,01	<0,01	<0,01	<0,01									
Guaviare	0,29	0,48	<0,01	<0,01	<0,01	0,01	0,06	0,34										
Huila	0,04	0,06	0,09	0,11	0,02	0,01	0,07	0,02	<0,01	<0,01	<0,01	<0,01	<0,01	<0,01	<0,01	<0,01	<0,01	
La Guajira	<0,01	<0,01	<0,01	<0,01	<0,01	<0,01	<0,01	<0,01	<0,01	<0,01	0,03	<0,01	<0,01	0,04	0,04			
Magdalena	0,04	0,34	0,27	0,22	0,18	0,60	0,38	0,08	0,05	0,26	0,31	0,58	0,41	0,13	0,04	0,16		
Meta	<0,01	<0,01	<0,01	0,01	0,01	0,04	<0,01	<0,01	<0,01	<0,01	<0,01	<0,01	<0,01	<0,01	<0,01	<0,01	<0,01	
Nariño	0,27	0,05	0,01	0,01	0,01	<0,01	0,07	0,38	0,15	0,06	0,01	0,01	0,01	0,01	0,24	0,74		
Norte de Santander	0,03	0,02	0,10	0,05	<0,01	<0,01	<0,01	<0,01	<0,01	<0,01	<0,01	0,01	0,03	0,03	0,16	0,11	0,04	
Putumayo	0,05	<0,01	<0,01	<0,01	<0,01	<0,01	<0,01	<0,01	<0,01	<0,01	<0,01							
Quindío	0,50	0,16	0,02	<0,01	<0,01	<0,01	<0,01	<0,01	<0,01	<0,01	<0,01	<0,01	<0,01	0,05	0,03	0,02		
Risaralda	0,08	0,15	0,12	0,11	0,09	0,10	<0,01	<0,01	<0,01	<0,01	<0,01	<0,01	<0,01	0,03	<0,01	<0,01	<0,01	
San Andrés	<0,01	0,01	0,01	<0,01	<0,01	<0,01	<0,01	<0,01	<0,01	<0,01	<0,01	<0,01	<0,01	<0,01	<0,01	<0,01		
Santander	0,13	0,12	0,02	<0,01	<0,01	<0,01	<0,01	<0,01	<0,01	<0,01	<0,01	<0,01	<0,01	<0,01	<0,01	<0,01		
Sucre	<0,01	<0,01	<0,01	<0,01	<0,01	<0,01	<0,01	<0,01	<0,01	<0,01	<0,01	<0,01	<0,01	<0,01	<0,01			
Tolima	0,02	<0,01	<0,01	<0,01	<0,01	0,26	<0,01	<0,01	<0,01	<0,01	<0,01	<0,01	<0,01	<0,01	<0,01			
Valle del Cauca	0,34	0,52	0,19	0,95	0,28	0,30	0,05	0,26	0,70	0,94	0,44	0,34	0,12	0,13	0,33	0,51	0,71	
Vaupés	0,02	<0,01	<0,01	<0,01	<0,01	<0,01	<0,01	<0,01	<0,01	<0,01	<0,01							
Vichada	<0,01	<0,01	<0,01	<0,01	<0,01	<0,01	<0,01	<0,01	<0,01									

* Se presentan los valores de p de la prueba de razón del logaritmo de la verosimilitud.

Dado que el 1° de junio de 2020 se decretó una flexibilización del confinamiento que buscaba reactivar la economía y ello implicaba una mayor probabilidad de transmisión del SARS-CoV-2, se analizaron los datos antes y después de esa fecha. Los resultados resumidos en el cuadro 2 sugieren que Antioquia, Nariño y Tolima tuvieron una buena contención, dado que la calidad del dato de segundos dígitos cumplieron con la distribución de Benford, y una adecuada vigilancia en salud pública después de la apertura económica iniciada el 1° de junio. Los departamentos de Cauca, Cesar, La Guajira y Norte de Santander lograron una buena calidad en los datos después de la apertura económica, pese a que antes no cumplían la ley de Benford; esto sugiere que se mejoró la vigilancia, lo cual suele ser el resultado de la preparación durante la contención. Bolívar, Cundinamarca y Sucre tuvieron una buena vigilancia en salud pública durante la contención, pero después de la apertura económica la calidad bajó, lo que sugiere que la capacidad instalada fue superada por la pandemia.

**Cuadro 2:** Cumplimiento de la ley de Benford complementario de los reportes de casos de COVID-19 en Colombia (casos acumulados)

Entidad territorial	Hasta 31 de mayo de 2020	Desde el 1° de junio de 2020 Primer dígito^¥^								
	Segundo dígito^*^	1	2	3	4	5	6	7	8	9
Amazonas	<0,01	<0,01	<0,01	<0,01	<0,01	<0,01	<0,01	<0,01	0,02	0,02
Antioquia	0,10	0,21	0,79	0,75	0,29	0,70	0,14	0,07	1,00	0,45
Arauca	ND	0,57	0,50	<0,01	0,22	0,70	0,83	<0,01	0,81	0,20
Atlántico	0,75	<0,01	0,42	0,34	0,11	<0,01	<0,01	0,38	0,09	0,45
Bolívar	0,92	0,11	0,03	0,08	0,60	0,45	0,22	0,81	1,00	1,00
Boyacá	0,15	<0,01	<0,01	0,01	0,29	0,12	1,00	0,82	0,24	0,45
Caldas	<0,01	0,37	<0,01	0,75	<0,01	0,12	0,02	0,82	0,09	0,45
Caquetá	0,01	<0,01	<0,01	0,43	0,29	0,17	0,83	0,04	0,81	0,45
Casanare	<0,01	0,02	0,01	<0,01	0,38	0,56	0,01	0,38	0,15	0,32
Cauca	<0,01	0,37	0,50	0,16	0,72	1,00	0,09	0,82	0,24	0,13
Cesar	<0,01	0,43	0,17	1,00	0,16	0,17	1,00	0,26	0,81	1,00
Chocó	0,07	<0,01	0,01	<0,01	0,03	0,12	0,09	0,04	0,24	1,00
Córdoba	0,04	<0,01	0,79	0,34	0,86	1,00	0,22	0,82	0,81	0,45
Cundinamarca	0,43	<0,01	0,68	0,53	0,38	0,12	1,00	0,82	0,63	0,80
Guainía	ND	<0,01	0,02	<0,01	0,01	<0,01	0,29	0,01	0,09	0,80
Guaviare	ND	0,91	0,48	0,19	0,58	1,00	0,03	1,00	1,00	0,80
Huila	<0,01	<0,01	0,01	<0,01	0,16	0,70	0,22	0,82	0,09	0,80
La Guajira	<0,01	0,82	0,22	0,75	0,29	0,85	1,00	0,38	0,63	0,80
Magdalena	0,10	0,26	0,17	0,04	0,60	0,25	1,00	0,01	0,34	0,80
Meta	<0,01	<0,01	0,42	0,21	0,22	0,45	0,53	0,82	1,00	0,01
Nariño	0,97	0,37	1,00	0,75	1,00	1,00	0,53	1,00	0,81	0,62
Norte de Santander	<0,01	0,37	0,59	0,43	0,38	0,84	1,00	0,66	0,48	0,80
Putumayo	ND	<0,01	0,06	0,53	0,08	0,01	0,02	0,38	0,24	0,32
Quindío	0,21	<0,01	0,79	0,04	0,38	0,45	0,83	0,18	0,24	0,45
Risaralda	<0,01	<0,01	0,34	0,16	0,48	0,05	0,41	1,00	0,63	0,80
San Andrés	<0,01	0,82	<0,01	0,27	0,86	0,12	0,02	0,04	0,09	0,13
Santander	<0,01	0,02	0,79	0,34	0,38	0,85	1,00	0,82	0,81	1,00
Sucre	0,39	0,91	0,79	0,01	0,72	0,56	0,68	0,26	0,15	0,62
Tolima	0,10	0,43	0,34	0,43	0,86	0,33	0,68	0,50	0,09	0,45
Vaupés	<0,01	0,31	<0,01	<0,01	<0,01	0,03	<0,01	<0,01	0,02	0,02
Vichada	ND	<0,01	0,68	<0,01	0,03	0,70	0,83	0,04	0,09	0,13

* Valores de p de la prueba de razón del logaritmo de la verosimilitud

^¥^ Valores p de la prueba de ji al cuadrado

ND: sin datos suficientes debido al inicio tardío de la pandemia

Los datos de la letalidad y la tasa por 100.000 habitantes se presentan en el cuadro 3. Es notorio que los departamentos con mayor letalidad son Córdoba, Norte de Santander, Magdalena, Putumayo y Atlántico, en tanto que los de menor letalidad son Vaupés, Guaviare, San Andrés y Providencia, Vichada y Risaralda. Al incorporar estos datos a los tres grupos generados con base en el cumplimiento de la ley de Benford, se obtuvo la clasificación del desempeño de la calidad del dato de la vigilancia en salud pública de los entes territoriales colombianos (cuadro 4).

**Cuadro 3:** Letalidad entre individuos diagnosticados con infección por SARS-CoV-2 en los territorios colombianos durante los primeros seis meses de pandemia

Entidad	Letalidad (%)
Córdoba	6,741
Norte de Santander	5,512
Magdalena	5,507
Putumayo	4,559
Atlántico	4,524
La Guajira	4,463
Sucre	4,203
Santander	4,113
Amazonas	4,105
Valle del Cauca	3,737
Chocó	3,688
Nariño	3,676
Caquetá	3,309
Quindío	3,051
Cauca	2,997
Bolívar	2,793
Cesar	2,792
Cundinamarca	2,784
Huila	2,668
Bogotá, D. C.	2,597
Tolima	2,525
Meta	2,284
Casanare	2,206
Antioquia	2,132
Boyacá	2,019
Caldas	1,952
Guainía	1,923
Arauca	1,856
Risaralda	1,769
Vichada	1,754
San Andrés	1,145
Guaviare	0,358
Vaupés	0,294

**Cuadro 4:** Clasificación del desempeño de la calidad del dato de la vigilancia en salud pública de las entidades territoriales colombianas durante los primeros 180 días de la pandemia de COVID-19

Desempeño	Entidad
Alto	Antioquia
	Tolima
	Bogotá
	Nariño
	Valle del Cauca
Intermedio	Cesar
	Cauca
	La Guajira
	Norte de Santander
Bajo	Vaupés
	Guaviare
	San Andrés
	Vichada
	Risaralda
	Arauca
	Guainía
	Caldas
	Boyacá
	Casanare
	Meta
	Huila
	Cundinamarca
	Bolívar
	Quindío
	Caquetá
	Chocó
	Amazonas
	Santander
	Sucre
	Atlántico
	Putumayo
	Magdalena
	Córdoba

El orden lo determina la calidad del alto y la letalidad, dentro de cada categoría

## Discusión

El presente estudio de la calidad del dato de la vigilancia en salud pública presenta los resultados hasta el 1° de septiembre del desempeño de la vigilancia en salud pública de COVID-19 en los departamentos colombianos. Lo primero que resalta es que el desempeño ha sido variado, con casos muy destacables y otros que no. El proceso de agregación ponderado por el número de casos reportados en cada ente territorial, encabezado por Bogotá y el Valle del Cauca, que suman entre los dos cerca del 38 % de los casos del total nacional, favoreció que el resultado global del desempeño nacional sea bueno. Con este efecto de la agregación de datos se puede entender que la vigilancia, como muchos temas de salud pública, conceptualmente tiene múltiples niveles que están interconectados.

Análisis como el que aquí se presenta han tenido gran acogida durante la pandemia de COVID-19. Es así como varios estudios han usado la ley de Benford para detectar posibles fraudes en los datos de la pandemia ^19^^,^^20^, establecer las características comunes de los países que no cumplen la ley de Benford ^21^ e, incluso, usar la distribución de Benford como criterio para definir si hay "aplanamiento" o no de la curva epidémica ^22^. En todos estos casos, los autores refieren que la objetividad del método es su mayor fortaleza.

En conclusión, los hallazgos de este estudio sugieren que Antioquia, Tolima, Bogotá, Nariño y Valle del Cauca tuvieron los mejores desempeños en cuanto a la calidad de los datos en los sistemas de vigilancia en salud pública. En futuros estudios se podrá completar la evaluación revisando otros indicadores sanitarios y sociales que muestren la complejidad del manejo de la pandemia. Dado que este estudio evaluó uno de los atributos de la vigilancia en salud pública, debe complementarse con la evaluación de los otros: la sensibilidad, el valor predictivo positivo, la flexibilidad, la aceptabilidad y la simplicidad, entre otros, pero en especial, del principio de utilidad ^23^. Estos hallazgos podrán contrastarse con aquellos alcanzados mediante otros indicadores, entre ellos, el número necesario de personas que deben diagnosticarse (número de pruebas por caso positivo), cuyo número mínimo aceptable es 20; el rastreo de los contactos evaluado mediante la proporción de casos relacionados con un caso previo que se detecte, y el aislamiento efectivo de los casos y contactos a partir de dicho rastreo ^24^.
